# Speaking up or remaining silent about patient safety concerns in rehabilitation: A cross‐sectional survey to assess staff experiences and perceptions

**DOI:** 10.1002/hsr2.631

**Published:** 2022-04-28

**Authors:** Andrea Niederhauser, David L. B. Schwappach

**Affiliations:** ^1^ Swiss Patient Safety Foundation Zurich Switzerland; ^2^ Institute of Social and Preventive Medicine (ISPM) University Bern Bern Switzerland

**Keywords:** communication, hospitals, rehabilitation, organizational culture, patient safety, surveys and questionnaires

## Abstract

**Background and Aims:**

Patient safety incidents may be prevented if healthcare workers speak up to voice their concerns when they observe hazardous clinical situations. This study aims to investigate the frequency of speaking up and healthcare workers' perception of organizational climate in rehabilitation clinics.

**Methods:**

An online survey was conducted in five rehabilitation clinics. An existing survey instrument (Speaking Up About Patient Safety Questionnaire [SUPS‐Q]) was adapted for this purpose. The instrument includes items on self‐reported speak‐up‐related behavior (perceived safety concerns, withholding voice, and speaking up), anticipated speak‐up behavior, barriers to speaking up, and speak‐up‐related climate measures (psychological safety, encouraging environment, and resignation). Data analysis included descriptive statistics, one‐way analysis of variance for differences between groups, multiple regression, and measures for validity and reliability of the scales.

**Results:**

Four hundred seventy‐one individuals participated in the survey (response rate of 32%). In the 4 weeks preceding the survey, 81% of respondents had specific concerns about patient safety, 83% performed speak up and 41% remained silent in one or more instances. Expected differences between professional groups were confirmed, but surprisingly, we found no effect of hierarchical level on speaking up behavior and perception of the speak‐up climate. Factors that most frequently prevented healthcare workers from speaking up were ineffectiveness (38%), presence of patients (26%), and unpredictability of the actor's reaction (25%). The psychometric evaluation of the adapted SUPS‐Q showed acceptable results for validity and reliability.

**Conclusions:**

Healthcare workers in rehabilitation clinics frequently perceive safety concerns. The study underlines the importance of promoting a culture of safety and speaking up. The short survey instrument SUPS‐Q can be used by rehabilitation clinics to initiate discussions related to facilitators and barriers to speaking up and to identify areas for improvement within the organization.

## 1 INTRODUCTION

In clinical care, healthcare workers (HCWs) often encounter situations, which raise concerns about patient safety or require clarification.^1^, ^2^ In these situations, open communication is crucial to prevent errors from reaching the patient and causing harm.^3^, ^4^, ^5^ The act of assertively voicing concerns, asking a question, or stating an opinion when one becomes aware of a hazardous clinical situation has been defined as “speaking up.”^1^, ^6^, ^7^ In addition to avoiding adverse events, speaking up in critical situations can prevent colleagues from making mistakes with potentially serious emotional consequences.^8^ Addressing safety concerns can also launch individual and organizational learning processes that help ensure that future patients and HCWs are not exposed to the same risks.^9^

Despite a strong motivation to protect patients from harm, many HCWs have encountered situations in which they found it challenging to speak up or where they even decided to withhold their concerns despite the potential risks.^2^, ^10^, ^11^ Different factors influence the decision to speak up or remain silent. Personality traits, educational background, and previous experiences of speaking up and its consequences factor in, as do organizational factors such as hierarchical structures and visible organizational and leadership support. The decision to speak up is also influenced by contextual factors such as clarity or ambiguity of the clinical situation and the perceived severity of harm to the patient.^6^ While raising safety concerns is usually associated with positive outcomes (i.e., preventing physical harm to patients), speaking up can also come with costs to the individual (e.g., negative response from others) and is thus antecedent by a personal trade‐off between presumed benefits and potential risks.^7^, ^11^

Effective team communication is crucial for the safe provision of rehabilitation care. A review of interprofessional team communication in rehabilitation found that teams with strong communication demonstrated shared values and mutual trust and respect, which includes the ability to openly express opinions during clinical discussions.^12^ It is, therefore, reasonable to assume that patient safety in rehabilitation clinics can be enhanced by encouraging and promoting speaking up in care teams. To our knowledge, however, speaking up has not yet been the subject of research in this healthcare setting.

The “Speaking Up About Patient Safety Questionnaire” (SUPS‐Q) is a short survey instrument developed for acute care hospitals. It allows organizations to assess staff behaviors, experiences, and perceptions related to speaking up.^13^ With this study, we aimed to adapt and pilot the SUPS‐Q in rehabilitation clinics, to gain insights as to how often HCWs perceive safety concerns, how often they chose to speak up or remain silent, and how they perceive organizational climate relevant for speaking up in their organization.

## 2 METHODS

The SUPS‐Q has been validated for use in acute care hospitals.^13^ To make it accessible to other healthcare settings, we conducted two studies with identical designs to adapt the instrument for inpatient rehabilitation and psychiatric hospitals. In this present paper, we report results for the adaptation of inpatient rehabilitation clinics. Results for the adaptation of psychiatric hospitals have recently been reported.^14^

### 2.1 Survey instrument

In the first section of the SUPQ‐Q, three scales with a total of 11 items assess respondents' self‐reported perceived safety concerns and frequency of withholding voice and speaking up. All items are rated on a 5‐point scale from “*never*” (0 times in the past 4 weeks) to “*very often*” (>10 times in the past 4 weeks). Higher frequencies of past behaviors result in higher mean scale values. The second section includes one multiple‐choice item covering six predefined barriers to speaking up. The third section surveys respondents' perception of speak‐up‐related climate at their workplace. The 11 items are organized into three subscales addressing psychological safety (five items), encouraging environment (three items), and resignation (three items). All items are rated on a 7‐point Likert scale, where the margins were labeled “*strongly disagree*” and “*strongly agree*.” Higher mean scale values indicate higher levels of perceived psychological safety, higher levels of perceiving an encouraging work environment, and higher levels of resignation towards speaking up. Lastly, the questionnaire includes a vignette to assess the respondents' anticipated behavior. After reading a hypothetical situation, respondents are asked to rate the realism of the situation, the expected risk for patient harm, their own likelihood to speak up, and their discomfort with speaking up in such a situation on a 7‐point response scale.

Fourteen health professionals with various backgrounds were invited to join a working group to discuss the necessary changes to make the SUPS‐Q applicable to rehabilitation clinics. The group suggested some changes to the wording of questions and response items to make them more comprehensible for rehabilitation staff. However, no items from the original questionnaire were removed or altered in content and no new items were added. There were some controversies over the vignette. While some group members judged the situation described in the standard SUPS‐Q version (a missed hand hygiene before wound inspection during daily rounds) to be realistic, others suggested that a missed hand hygiene situation related to a patient placed in isolation would more adequately depict safety concerns in their area of work. As no consensus could be reached, it was decided to test both options (see Table 4 for wording). Participants randomly received a questionnaire with either vignette 1 or 2.

### 2.2 Study population and procedures

The online survey was administered in five rehabilitation clinics in Switzerland (convenience sample) in October–November 2018. Participating clinics ranged in size from 34 beds to 250 beds. All clinics provided inpatient services after hospital discharge. All HCWs with direct patient contact were invited to participate in the survey. Local study coordinators at each site were responsible for identifying eligible staff members and sending an e‐mail invitation with the link to the anonymous online questionnaire. HCWs could choose not to participate and survey completion was considered informed consent. Participants were informed that the results of the survey would be used for research purposes to validate the adapted version of the SUPS‐Q and that they would be notified about the results for their respective clinics. The online survey could be accessed for 4 weeks, one or two reminders were sent at each site.

### 2.3 Data analysis

For all items, scales, and subscales, descriptive statistics were calculated. Missing values were excluded pairwise. To measure internal consistency, Cronbach's *α* was calculated for the scales and subscales. A value of >0.7 was considered to indicate acceptable consistency. Convergent and divergent validity was assessed by examining the correlation between items and scale scores. A confirmatory factor analysis (CFA) using maximum likelihood estimation methods was conducted to test the defined three‐factor structure of the speaking up climate data. Model fit was tested using comparative fit index (CFI) (acceptable fit 0.90–0.95, good fit ≥0.95), root mean square error of approximation (RMSEA) (good fit ≤0.06) and standardized root mean squared error (SRMR) (good fit ≤0.08).^15^, ^16^, ^17^ One‐way analysis of variance was used to determine differences in mean scores between professional groups (nurses, physicians, therapists) and levels of hierarchy (high/low). Respondents' managerial function (yes/no) was used to determine the level of hierarchy. We expected climate scores to be higher for physicians and staff with managerial function, as compared to nurses and staff without managerial function, respectively.^13^ In addition, we hypothesized that staff members frequently caring for patients requiring acute medical care (e.g. wound care, high‐risk medication) had higher levels of safety concerns and withholding voice than their colleagues. Necessity to speak up is often perceived in situations where norms or standards are violated.^1^ It seems likely that with the increased provision of acute medical care in rehabilitation clinics, the number of rules and standards related to high‐risk care increase, and concerns for safety gain relevance. Lastly, we used multiple regression to analyze the relationship between anticipated likelihood to speak up and perceived risk of harm, hierarchical level, and professional group.^18^, ^19^, ^20^ For all analyses, *p* < 0.05 was considered statistically significant. All analyses were performed with Stata 14.1.

## 3 RESULTS

Of the 1494 eligible HCWs, 471 (32%) participated in the survey (range between facilities: 26%—49%). Table 1 summarizes the characteristics of the study sample.

**Table 1:** Details of the study sample (*n* = 471).

		*n*	%
Facility	A (34 beds)	72	15
	B (96 beds)	94	20
	C (250 beds)	103	22
	D (244 beds)	123	26
	E (170 beds)	79	17
Gender	Female	369	78
	Male	79	17
	Missing	23	5
Age (years), mean (SD)		39.8	(12.0)
	Missing	85	18.0
Profession	Nurse	199	42
	Physician	24	5
	Therapist^a^	134	28
	Other	76	16
	Missing	38	8
In education	Yes	32	7
	No	427	91
	Missing	12	3
Hierarchical level	High	115	24
	Low	322	68
	Missing	12	7
Weekly work hours in patient care (h)	<10	52	11
	10–24	117	25
	25–39	162	34
	>40	110	23
	Missing	30	6
Years working in hospital, mean (SD)		7.2	(6.8)
	Missing	95	20
Frequency of care for patients requiring acute medical care	Rarely or never	150	32
	Frequently or sometimes	300	64
	Missing	21	4

^a^ The category “therapist” includes: physical therapist, occupational therapist, other medical‐therapeutic staff, therapy expert.

### 3.1 Psychometric evaluation of survey instrument

For the behavior‐related items, Cronbach's *α* indicated good internal consistency for all three scales (Table 2). All items had a correlation coefficient with the score of their own scale greater than 0.6 and a correlation coefficient with the score of their own scale greater than with other scales. In regard to the climate‐related scale, all but two of the 11 items had a correlation coefficient with the score of their own subscale greater than 0.4 and a correlation coefficient with the score of their own subscale greater than with other subscales. Cronbach's *α* shows good internal consistency of the total climate scale and moderate to good internal consistency for the three subscales. Factor loadings were high (>0.6) for seven items and moderate (>0.4) for four items. Results of the confirmatory factor analysis revealed mixed results. The CFI showed good fit (0.95), the RMSEA unsatisfactory fit (0.079), and the SRMR good fit (0.048).

**Table 2:** Frequencies of reporting perceived concerns, withholding voice, and speaking up within the last 4 weeks (Items translated from German original).

*In everyday work, it sometimes happens that things go wrong and risks to patients arise. This could be as a result of medication error, omitted hand hygiene, or noncompliance with standards. Over the last 4 weeks, how frequently…*					
	***N*(%)**				
	**Never (0 times)**	**Rarely (1–2 times)**	**Sometimes (3–5 times)**	**Often (6–10 times)**	**Very often (>10 times)**
Perceived concerns (Cronbach's *α* = 0.83)					
*… have you had specific concerns about patient safety?*	88 (19)	184 (39)	135 (29)	42 (9)	19 (4)
*… have you observed an error which—if uncaptured—could be harmful to patients?*	148 (32)	202 (43)	94 (20)	20 (4)	2 (<1)
*… have you noticed that your workplace colleagues* a *didn't follow important patient safety rules, intentionally or unintentionally?*	160 (34)	162 (35)	96 (21)	42 (9)	7 (2)
Withholding voice (Cronbach's *α* = 0.82)					
*… did you choose not to bring up your specific concerns about patient safety?*	274 (59)	129 (28)	43 (9)	12 (3)	3 (1)
*… did you keep ideas for improving patient safety in your area of work to yourself?*	266 (57)	121 (26)	54 (12)	18 (4)	4 (1)
*… did you remain silent when you had information that might have prevented a safety incident?*	357 (77)	84 (18)	17 (4)	4 (1)	1 (<1)
*… did you not address a colleague* a *if he/she didn't follow, intentionally or unintentionally, important patient safety rules?*	277 (60)	123 (27)	46 (10)	10 (2)	5 (1)
Speaking up (Cronbach's *α* = 0.90)					
*… did you bring up specific concerns about patient safety?*	79 (17)	191 (41)	127 (27)	52 (11)	13 (3)
*… did you address an error which—if uncaptured—could be harmful to patients?*	92 (20)	178 (39)	116 (25)	62 (13)	13 (3)
*… did you address a colleague* a *when he/she didn't follow, intentionally or unintentionally, important patient safety rules?*	148 (32)	170 (37)	94 (21)	34 (7)	11 (2)
*… did you prevent an incident from occurring as a consequence of bringing up specific concerns about patient safety?*	174 (42)	143 (34)	72 (17)	24 (6)	4 (1)

^a^ Colleagues is defined as “across professional groups and hierarchies.”

### 3.2 Speak‐up behavior

In the 4 weeks preceding the survey, 81% of respondents had perceived specific concerns about patient safety in one or more instances. During the same time span, most participants (83%) had voiced their concerns at least once. Yet, many also stated that there had been at least one or more instances where they had decided not to bring up concerns (41%), keep important information (23%), or not address rule violations (40%) (Table 2). Comparing mean scale sores, there were no significant differences in the frequency of perceiving concerns between physicians, nurses, and therapists (mean_nurs_ 2.3, mean_phys_ 1.9, mean_ther_ 2.2; *p* = 0.06). Nurses and therapists had higher mean scale scores for withholding voice than physicians (mean_nurs_ 1.6, mean_phys_ 1.1, mean_ther_ 1.6; *p* = 0.003). Among the three professional groups, nurses had the highest mean scale scores for speaking up (mean_nurs_ 2.5, mean_phys_ 1.9, mean_ther_ 2.2; *p* < 0.001). Contrary to expectations, we found no differences in mean scale scores for perceiving concerns (mean 2.2 vs. 2.1, *p* = 0.33), withholding voice (mean 1.4 vs. 1.5, *p* = 0.15) and speaking up (mean 2.3 vs. 2.2, *p* = 0.35) for staff of higher and lower levels of hierarchy. Respondents who frequently provide acute medical care had significantly higher mean scale scores for perceiving concerns (mean 2.3 vs. 1.8, *p* < 0.001), withholding voice (mean 1.6 vs. 1.3, *p* < 0.001), and speaking up (mean 2.5 vs. 1.8, *p* < 0.001) as compared to those rarely caring for patients requiring acute medical care.

### 3.3 Speak‐up climate

Total climate scores were significantly lower for nurses and therapists compared to physicians (Table 3). In particular, their scores were less positive for items assessing resignation towards speaking up. Climate ratings between hierarchical levels did not significantly differ for any individual item (data not shown), nor for the total climate score (mean 4.9 vs. 4.9, *p* = 0.76). For staff frequently caring for patients requiring acute medical care, climate scores were significantly lower for 6 out of 11 items (data not shown) and for the total climate score (mean 4.8 vs. 5.2, *p* < 0.001), as compared to their colleagues.

**Table 3:** Mean (SD) responses to climate survey items by professional group.

	All^a^, ^b^				Nurses			Physicians		Therapists				*p* Value^c^	
Psychological safety for speaking up (Cronbach's *α* = 0.80)															
*I can rely on my colleagues* d *whenever I encounter difficulties in my work*.	5.3 (1.6)				5.2 (1.6)			5.5 (1.6)		5.2 (1.7)				0.69	
*I can rely on my supervisor whenever I encounter difficulties in my work*.	5.8 (1.7)				5.7 (1.6)			5.6 (2.1)		5.9 (1.6)				0.71	
*The culture in my area of work makes it easy to speak up about patient safety concerns*.	4.9 (1.8)				4.8 (1.8)			5.8 (1.4)		4.7 (1.9)				0.02	
*My colleagues* d *react appropriately when I speak up about my concerns about patient safety*.	5.1 (1.6)				5.2 (1.4)			5.1 (1.6)		5.0 (1.6)				0.24	
*My supervisors react appropriately when I speak up about my patient safety concerns*.	5.6 (1.6)				5.6 (1.5)			5.3 (1.8)		5.6 (1.5)				0.70	
Encouraging environment for speaking up (Cronbach's *α* = 0.65)															
*In my area of work, I observe others speaking up about their patient safety concerns*.	4.8 (1.8)				4.9 (1.6)			5.0 (1.8)		5.0 (1.8)				0.81	
*I am encouraged by my colleagues* d *to speak up about patient safety concerns*.	4.4 (1.9)				4.6 (1.9)			4.8 (2.0)		4.2 (1.8)				0.06	
*I am encouraged by my supervisors to speak up about patient safety concerns*.	4.3 (2.0)				4.7 (1.9)			4.2 (2.3)		4.1 (1.8)				0.04	
Resignation towards speaking up (Cronbach's *α* = 0.74)															
*When I have patient safety concerns it is difficult to bring them up*.^e^	2.5 (1.7)				2.5 (1.6)			1.5 (0.9)		2.8 (1.7)				0.001	
*Having to remind staff of the same clinical standards again and again is frustrating*.^e^	4.2 (2.2)				4.2 (2.1)			2.4 (1.6)		4.7 (2.1)				<0.001	
*Sometimes I become discouraged because nothing changes after expressing my patient safety concerns*.^e^	3.6 (2.1)				3.8 (2.1)			2.0 (1.5)		4.1 (2.0)				<0.001	
Total speak‐up climate score (Cronbach's *α* = 0.83)	4.9 (1.1)				4.9 (1.1)			5.4 (1.1)		4.8 (1.1)				0.03	

^a^ All ratings measured on a 7‐point scale from “strongly disagree” (1) to “strongly agree” (7).

^b^ Including other professions (*n* = 76) and respondents with missing values on profession (*n* = 38).

^c^ Significance level of analysis of variance for differences in mean scores between nurses, therapists, and physicians.

^d^ Colleagues is defined as “across professional groups and hierarchies.”

^e^ Negatively worded items are reverse coded for the total score.

### 3.4 Barriers to speaking up

**Figure 1:**
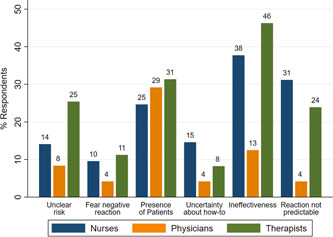
Relative frequencies of self‐reported barriers to speaking up, by professional group.

The most frequently reported barrier to speaking up was ineffectiveness (38%), followed by the presence of patients (26%) and the unpredictable reaction of the person causing concerns (25%). The assessment of the barriers differed in part between professional groups (Figure 1). Ineffectiveness was reported more frequently as an important barrier by both nurses and therapists as compared to physicians (*p* = 0.006), as was the unpredictable reaction of the person causing concerns (*p* = 0.01). For therapists, the unclear risk in a situation represented more often a barrier than for nurses and physicians (*p* = 0.01).

### 3.5 Anticipated behavior

Two hundred thirty‐one (49%) participants responded to vignette 1 and 240 (51%) responded to vignette 2. In both vignettes, scores for the anticipated likelihood of speaking up in the given situation differed significantly between professional groups and hierarchical levels (Table 4). For vignette 1, regression analysis showed that professional group, higher hierarchical status, and perceiving a high potential for harm in the situation were positively associated with the self‐reported likelihood to speak up (physicians compared to nurses: unstandardized coefficient 0.40, *p* = 0.57/therapists compared to nurses: unstandardized coefficient −1.05, *p* = 0.001, hierarchical level: unstandardized coefficient 0.85, *p* = 0.02, perceived risk of harm for the patient: unstandardized coefficient 0.24, *p* = 0.03). Similarly, for vignette 2, professional group (physicians compared to nurses: unstandardized coefficient 1.16, *p* = 0.03/therapists compared to nurses: unstandardized coefficient −0.59, *p* = 0.047), higher hierarchical status (unstandardized coefficient 0.64, *p* = 0.048) and perceiving a high risk of harm for the patient (unstandardized coefficient 0.36, *p* = 0.003) were positively associated with the self‐reported likelihood to speak up.

**Table 4:** Mean vignette ratings by subgroups.

	Vignette ratings,^a^ mean (SD)			
	Situation is realistic	Perceived risk of harm	Likelihood to speak up	Discomfort with speaking up
Vignette 1 (*n* = 231)				
*You are on a daily round as part of an interdisciplinary team (physicians, nurses, therapists). The senior physician greets a patient with a handshake. He wants to examine the patients' wounds. However, the senior physician does not use gloves and he did not disinfect his hands again*.				
Total	3.7 (2.1)	5.3 (1.5)	4.3 (2.0)	4.0 (2.1)
Professional group, *p* b	<0.001	0.002	0.003	0.005
Nurses	4.4 (1.9)	5.3 (1.4)	4.6 (2.1)	4.0 (2.2)
Physicians	2.3 (2)	3.5 (1.7)	5.0 (2.2)	2.5 (1.8)
Therapists	3.4 (1.8)	5.4 (1.3)	3.6 (1.7)	4.6 (1.7)
Hierarchical level, *p* b	0.65	0.54	0.004	0.001
Low	3.7 (2.0)	5.3 (1.4)	4.1 (2.0)	4.2 (2.1)
High	3.8 (2.3)	5.1 (1.8)	5.0 (2.0)	3.2 (2.0)
Acute medical care, *p* b	<0.001	0.66	0.99	0.23
Rarely	3.1 (2.0)	5.2 (1.5)	4.3 (2.1)	3.8 (2.0)
Frequently	4.1 (2.0)	5.3 (1.5)	4.3 (2.0)	4.1 (2.1)
Vignette 2 (*n* = 240)				
*You are on a daily round as part of an interdisciplinary team (doctors, nurses, therapists) with a patient who had been placed in isolation due to a Norovirus infection. The senior physician greets the patient with a handshake. He does not wear gloves. When entering the next patient's room, you notice that the senior physician did not disinfect his hands again*.				
Total	3.5 (2.0)	6.1 (1.3)	5.3 (2.0)	3.5 (2.2)
Professional group, *p* b	0.01	0.38	0.01	<0.001
Nurses	4.0 (2.0)	6.1 (1.2)	5.5 (1.9)	3.4 (2.2)
Physicians	2.9 (2.1)	5.8 (1.2)	6.7 (0.7)	1.9 (1.4)
Therapists	3.2 (1.9)	6.2 (1.1)	5.0 (2.0)	4.1 (1.9)
Hierarchical level, *p* b	0.13	0.70	0.008	0.005
Low	3.4 (2.0)	6.0 (1.3)	5.1 (2.1)	3.7 (2.1)
High	3.8 (2.1)	6.1 (1.2)	5.9 (1.7)	2.7 (2.1)
Acute medical care, *p* b	0.07	0.60	0.81	0.27
Rarely	3.1 (2.0)	6.0 (1.5)	5.3 (2.1)	3.2 (2.2)
Frequently	3.7 (2.1)	6.1 (1.2)	5.3 (2.0)	3.6 (2.2)

^a^ All ratings were measured on a 7‐point scale. Higher mean values indicate higher levels of realism of the situation, perceived risk of harm, anticipated likelihood to speak up, and discomfort with speaking up.

^b^ One way analysis of variance for differences in mean ratings between respondents of different professional groups and hierarchical levels.

## 4 DISCUSSION

In this study, we used an adapted version of the SUPS‐Q to assess staff perceptions and experiences with speaking up in rehabilitation clinics. We found that HCWs in rehabilitation clinics frequently observe errors or are concerned about incidents that threaten patient safety in their clinical practice. This indicates that there is a high potential for avoiding adverse events if HCWs are encouraged and feel free to speak up when noticing errors, unsafe behaviors, or violations of safety rules. Encouragingly, a majority of HCWs had already performed speak up when needed to prevent safety incidents. However, many participants also reported having remained silent in situations that might have warranted voicing concerns. The main barriers to speaking up were ineffectiveness, that is, expecting no productive response even after voicing concerns, the presence of patients, and an unpredictable reaction of parties involved. In our study, respondents provided moderate to high scores on items assessing the speak‐up climate in their organization. Participants indicated feeling comfortable voicing concerns without having to fear inappropriate reactions from colleagues and supervisors. However, they did not perceive strong encouragement from their environment to speak up and sometimes experience resignation when nothing changes after having voiced concerns.

Expected differences between professional groups were confirmed. Nurses speak up more frequently, but they also decide to remain silent more often than physicians. Qualitative studies suggest that nurses may be hesitant to speak up due to perceived lack of support, instances of being ignored, and experiences of being disrespected.^21^ The feeling of resignation can foster withholding voice. This is reflected in our results where ineffectiveness was the main barrier reported by nurses and therapists. Compared to acute care, rehabilitation care is characterized by greater involvement of therapeutical staff such as physical or occupational therapists. In our survey, we found that response patterns from therapists were comparable to those of nurses, that is, they speak up, but also withhold their voice frequently. Therapists oftentimes face clinical situations that require an intervention to prevent potential harm but may be insecure about the risk of harm or wary that their concerns may not be heard. By training and supporting them to effectively communicate within the care team, their role as important agents for patient safety can be acknowledged and fostered.

Surprisingly, we found no effect of hierarchical level on speaking up behavior and perception of speak‐up climate in our sample. Previous studies in the hospital setting have concluded that status asymmetry greatly affects speaking up behavior and perception of safety climate,^21^, ^22^ but these dynamics were not predominant in our sample. This may result from bias due to self‐selection of participants less susceptible to power dynamics. Greater proximity of leaders to clinical work, moderate‐sized teams, and stronger social skills among leaders in rehabilitation care may also help to explain our findings. More research is needed to understand how authority gradients affect speak‐up behavior in rehabilitation care as compared to acute care hospitals.

Finally, we found that HCWs frequently caring for patients requiring acute medical care had higher frequencies of safety concerns, speaking up and withholding voice, and less positive climate scores than their colleagues. This supports our hypothesis that speaking up is of high—and probably growing—relevance for HCWs in rehabilitation clinics. A study in Australia found that between 2007 and 2016, patients admitted to inpatient rehabilitation became more complex and dependent.^23^ The increased provision of acute medical care has implications for the delivery of rehabilitation services and the skills required of HCWs to provide these services. There is little evidence that rehabilitation clinics in Switzerland have seen similar changes in patient population, however, there are some reports suggesting that patients are discharged from acute to post‐acute care sooner and are admitted to rehabilitation clinics with an increased need for acute medical care. Statistical data shows that during the years 2000 and 2017, length of stay of elderly patients has decreased by 3.1 days in acute hospitals and by (additional) 3.2 days in rehabilitation clinics in Switzerland, indicating a trend toward “quicker and sicker” transfers.^24^, ^25^ It is conceivable that these changes have substantial implications for patient safety and should be examined in future studies to identify potential threats, as well as appropriate measures to address patient safety risks in rehabilitation.

An adapted version of the SUPS‐Q was piloted in this study. The advantages of this short instrument are that it can be self‐administered by rehabilitation clinics to evaluate speak‐up behavior and climate in their organization or to monitor changes over time. Clinics can use the survey results to identify areas for improvement and emphasize their commitment to an encouraging speak‐up environment. The disadvantages of the instrument are that it relies on self‐reported data and that it cannot capture the complexity of the individual decision‐making processes that characterize this act of communication. The psychometric evaluation of the adapted SUPS‐Q showed acceptable results for validity and reliability. The results confirmed expected differences in scores between professional groups, but, surprisingly, not for hierarchical groups. Convergent and divergent analyses showed high correlations between items and subscale scores. All scales showed good internal consistency. The three‐factor structure of the speaking up climate data was moderately supported by the CFA. From these initial results, several issues for further improvement of the instrument emerge. Items could be reworded or replaced, and new items could be added to increase the internal reliability of scales. The factor structure of the climate scale could be revised based on exploratory factor analysis to potentially better fit the data from the rehabilitation setting. While all these options are straightforward and would probably lead to a more specific instrument for the rehabilitation setting, it would come at the cost of losing comparability with other settings. Our main intention with the adaption of the survey instrument was to provide a measure applicable to different settings to allow for system‐wide studies and inspection of similarities and differences. With this trade‐off in mind, we make two recommendations for future study and application. The instrument could be applied under stringent and strict design considerations in a sample of staff from various settings, for example, paying attention to selection and nonresponse bias. Results would confirm or rebut whether the instrument indeed performs poorer in nonacute settings and whether any adaptions could be made to account for this while retaining the generic character of the measures. Until this clarity has been reached, results could be analyzed on the single item level or the total climate scale rather than its subscales to avoid problems associated with scale reliabilities and factor structure. Results for the two vignettes tested for the adapted SUPS‐Q differed primarily in the level of the perceived risk of harm. However, for both vignettes, regression analysis confirmed the hypothesized associations between anticipated likelihood to speak up and risk of harm. In line with previous studies,^18^, ^19^, ^26^ we found that the personal perception of harm for the patient and the respondent's hierarchical status are strong predictors of the likelihood to speak up. Both vignettes are thus suitable to survey anticipated speak‐up behaviors and we cannot provide a conclusive recommendation for either scenario. Users of the adapted SUPS‐Q are encouraged to include the vignette that better represents a typical situation triggering safety concerns in their clinical context. Again, comparability with other settings may be an important criterion for this decision.

## 5 LIMITATIONS

The study has some limitations. The response rates ranged from low (26%) to good (47%) between the clinics. We cannot exclude selection bias in this study. Staff members' willingness to participate in the survey may have been affected by past positive or negative experiences of speaking up. As there was no data about nonrespondents available, we could not assess the representativeness of the study sample. We relied on self‐reported measures to examine the extent of speak‐up behavior among HCWs, making our results susceptible to response and recall bias. Single experiences of patient safety incidents may be overrepresented in individuals' memory, which could result in higher frequencies of reported behaviors. However, due to its spontaneous nature, it is difficult to objectively measure speak‐up behavior in practice. More importantly, withholding voice is a “nonbehaviour” not easily accessible to observation. New methodologies, such as analyzing verbatim transcripts of speak‐up interactions or designing simulation experiments have been proposed to overcome some of the methodological limitations of self‐reported data.^7^, ^27^

Finally, the results of the regression analysis may be subject to common methods bias (CMB) as both predictor and outcome variables were obtained within the same survey.^28^ We asked for only a few personal data and ensured participants their anonymity at various instances (invitation, start of the survey, etc.) to minimize social desirability bias as one source of CMB. Other recommended procedural remedies, for example, separating predictor and outcome variable measurements by a time lag, seem not appropriate in a vignette study design in which outcome and predictor ratings need to be organized closely around the vignette. The relevance of CMB has been questioned recently and ex post statistical control of CMB, such as marker variable approaches, have not been recommended for general application.^29^, ^30^

## 6 CONCLUSIONS

Using a previously validated short survey instrument adapted to the rehabilitation setting, we were able to demonstrate the importance of promoting a culture of safety and speaking up. We found that safety concerns occur frequently, but HCWs may be hesitant to voice their concerns in specific situations or are tired of not being heard. The increasing provision of acute medical care in rehabilitation clinics could accentuate the importance of speaking up to avoid rule violations and adverse events related to high‐risk care. The adapted survey instrument can be a useful tool for rehabilitation clinics to initiate discussions related to facilitators and barriers to speaking up and to identify areas for improvement within the organization. Future studies should focus on understanding the nature of safety concerns, the dynamics between professional and hierarchical groups, as well as cultural and contextual factors influencing decisions to speak up in rehabilitation clinics.

## AUTHOR CONTRIBUTIONS

**Andrea Niederhauser**: conceptualization; formal analysis; investigation; methodology; project administration; writing – original draft. **David L. B. Schwappach**: conceptualization; formal analysis; investigation; methodology; supervision; writing – review & editing.

## CONFLICTS OF INTEREST

The authors declare no conflicts of interest.

## TRANSPARENCY STATEMENT

The lead author (manuscript guarantor) affirms that this manuscript is an honest, accurate, and transparent account of the study being reported; that no important aspects of the study have been omitted; and that any discrepancies from the study as planned (and, if relevant, registered) have been explained.

## ETHICS STATEMENT

The study does not require full ethical review according to the Human Research Act, as confirmed by the Ethics Committee of the Canton of Zurich, Switzerland (BASEC‐Nr. Req‐2018‐00681).

## Data Availability

The data sets used and/or analyzed during the current study are available from the corresponding author on reasonable request.

## References

[1] Schwappach DLB , Gehring K . ‘Saying it without words’: a qualitative study of oncology staff's experiences with speaking up about safety concerns. BMJ Open. 2014;4:e004740. 10.1136/bmjopen-2013-004740 PMC4025461 24838725

[2] Martinez W , Lehmann LS , Thomas EJ , et al. Speaking up about traditional and professionalism‐related patient safety threats: a national survey of interns and residents. BMJ Qual Saf. 2017;26:869‐880. 10.1136/bmjqs-2016-006284 28442609

[3] Sutcliffe KM , Lewton E , Rosenthal MM . Communication failures: an insidious contributor to medical mishaps. Acad Med. 2004;79:186‐194. 10.1097/00001888-200402000-00019 14744724

[4] Leonard M . The human factor: the critical importance of effective teamwork and communication in providing safe care. Qual Saf Heal Care. 2004;13:i85‐i90. 10.1136/qshc.2004.010033 PMC1765783 15465961

[5] Greenberg CC , Regenbogen SE , Studdert DM , et al. Patterns of communication breakdowns resulting in injury to surgical patients. J Am Coll Surg. 2007;204:533‐540. 10.1016/j.jamcollsurg.2007.01.010 17382211

[6] Okuyama A , Wagner C , Bijnen B . Speaking up for patient safety by hospital‐based health care professionals: a literature review. BMC Health Serv Res. 2014;14:61. 10.1186/1472-6963-14-61 24507747 PMC4016383

[7] Noort MC , Reader TW , Gillespie A . Speaking up to prevent harm: A systematic review of the safety voice literature. Saf Sci. 2019;117:375‐387. 10.1016/j.ssci.2019.04.039

[8] Waterman AD , Garbutt J , Hazel E , et al. The emotional impact of medical errors on practicing physicians in the United States and Canada. Jt Comm J Qual Patient Saf. 2007;33:467‐476. 10.1016/S1553-7250(07)33050-X 17724943

[9] Jones A , Kelly D . Deafening silence? Time to reconsider whether organisations are silent or deaf when things go wrong. BMJ Qual Saf. 2014;23:709‐713. 10.1136/bmjqs-2013-002718 25015116

[10] Maxfield D , Grenny J , McMillan R , et al. Silence Kills: The Seven Crucial Conversations for Healthcare. VitalSmarts, L.C; 2005.

[11] Schwappach DL , Gehring K . Trade‐offs between voice and silence: a qualitative exploration of oncology staff's decisions to speak up about safety concerns. BMC Health Serv Res. 2014;14:303. 10.1186/1472-6963-14-303 25017121 PMC4105519

[12] Paxino J , Denniston C , Woodward‐Kron R , Molloy E . Communication in interprofessional rehabilitation teams: a scoping review. Disabil Rehabil. 2020;23:1‐17. 10.1080/09638288.2020.1836271 33096000

[13] Richard A , Pfeiffer Y , Schwappach DDL . Development and psychometric evaluation of the Speaking Up About Patient Safety Questionnaire. J Patient Saf. 2021;17:e599‐e606. 10.1097/PTS.0000000000000415 28858000

[14] Schwappach DLB , Niederhauser A . Speaking up about patient safety in psychiatric hospitals—a cross‐sectional survey study among healthcare staff. Int J Ment Health Nurs. 2019;28:1363‐1373. 10.1111/inm.12664 31609065 PMC6919932

[15] Bentler PM . Comparative fit indexes in structural models. Psychol Bull. 1990;107:238‐246. 2320703 10.1037/0033-2909.107.2.238

[16] Hu LT , Bentler PM . Cutoff criteria for fit indexes in covariance structure analysis: conventional criteria versus new alternatives. Struct Equ Model. 1999;6:1‐55. 10.1080/10705519909540118

[17] Hooper D , Coughlan J , Mullen MR . Structural equation modelling: guidelines for determining model fit. Electron J Bus Res Methods. 2008;6:53‐60.

[18] Schwappach DLB . Speaking up about hand hygiene failures: a vignette survey study among healthcare professionals. Am J Infect Control. 2018;46:870‐875. 10.1016/j.ajic.2018.02.026 29650487

[19] Schwappach DLB , Gehring K . Silence that can be dangerous: a vignette study to assess healthcare professionals' likelihood of speaking up about safety concerns. PLOS One. 2014;9:e104720. 10.1371/journal.pone.0104720 25116338 PMC4130576

[20] Lyndon A , Sexton JB , Simpson KR , Rosenstein A , Lee KA , Wachter RM . Predictors of likelihood of speaking up about safety concerns in labour and delivery. BMJ Qual Saf. 2012;21:791‐799. 10.1136/bmjqs-2010-050211 PMC3264837 22927492

[21] Morrow KJ , Gustavson AM , Jones J . Speaking up behaviours (safety voices) of healthcare workers: A metasynthesis of qualitative research studies. Int J Nurs Stud. 2016;64:42‐51. 10.1016/j.ijnurstu.2016.09.014 27684321

[22] Singer SJ , Falwell A , Gaba DM , Baker LC . Patient safety climate in US hospitals. Med Care. 2008;46:1149‐1156. 10.1097/MLR.0b013e31817925c1 18953225

[23] McKechnie D , Pryor J , Fisher MJ , Alexander T . Examination of the dependency and complexity of patients admitted to in‐patient rehabilitation in Australia. Aust Heal Rev. 2020;44:143‐152. 10.1071/AH18073 30654857

[24] Schweizerisches Gesundheitsobservatorium OBSAN . Aufenthaltsdauer in Akutspitälern. BFS—Medizinische Stat. der Krankenhäuser. 2018. Accessed February 3, 2020. https://www.obsan.admin.ch/de/indikatoren/aufenthaltsdauer-akutspitaelern

[25] Schweizerisches Gesundheitsobservatorium OBSAN . Aufenthaltsdauer in Rehabilitationseinrichtungen. BFS–Medizinische Stat. der Krankenhäuser. 2018. Accessed February 3, 2020. https://www.obsan.admin.ch/de/indikatoren/aufenthaltsdauer-rehabilitationseinrichtungen

[26] Lyndon A , Sexton JB , Simpson KR , et al. Predictors of likelihood of speaking up about safety concerns in labour and delivery. BMJ Qual Saf. 2012;21:791‐799. 10.1136/bmjqs.2010.050211 PMC3264837 22927492

[27] Noort MC , Reader TW , Gillespie A . Walking the plank: an experimental paradigm to investigate safety voice. Front Psychol. 2019;10:668. 10.3389/fpsyg.2019.00668 31001165 PMC6454216

[28] Podsakoff PM , MacKenzie SB , Lee JY , Podsakoff NP . Common method biases in behavioral research: a critical review of the literature and recommended remedies. J Appl Psychol. 2003;88:879‐903. 14516251 10.1037/0021-9010.88.5.879

[29] Conway JM , Lance CE . What reviewers should expect from authors regarding common method bias in organizational research. J Bus Psychol. 2010;25:325‐334. 10.1007/s10869-010-9181-6

[30] Schaller TK , Patil A , Malhotra NK . Alternative techniques for assessing common method variance: an analysis of the theory of planned behavior research. Organ Res Methods. 2015;18:177‐206. 10.1177/1094428114554398

